# Pre-Pregnancy Body Mass Index, Gestational Weight Gain, and Birth Weight: A Cohort Study in China

**DOI:** 10.1371/journal.pone.0130101

**Published:** 2015-06-26

**Authors:** Shaoping Yang, Anna Peng, Sheng Wei, Jing Wu, Jinzhu Zhao, Yiming Zhang, Jing Wang, Yuan Lu, Yuzhen Yu, Bin Zhang

**Affiliations:** 1 Department of Primary Guidance, Wuhan Women and Children Health Care Center, Wuhan, Hubei Province, 430015, China; 2 Key Laboratory of Environment and Health, Ministry of Education & Department of Epidemiology and Biostatistics, School of Public Health, Tongji Medical College, Huazhong University of Science and Technology, Wuhan, China; 3 Maternal and Children Health Care Department of Districts of Wuhan, Wuhan, China; University of Bremen, GERMANY

## Abstract

**Objective:**

To assess whether pre-pregnancy body mass index (BMI) modify the relationship between gestational weight gain (GWG) and child birth weight (specifically, presence or absence of low birth weight (LBW) or presence of absence of macrosomia), and estimates of the relative risk of macrosomia and LBW based on pre-pregnancy BMI were controlled in Wuhan, China.

**Methods:**

From June 30, 2011 to June 30, 2013. All data was collected and available from the perinatal health care system. Logistic regression models were used to estimate the independent association among pregnancy weight gain, LBW, normal birth weight, and macrosomia within different pre-pregnancy BMI groups. We built different logistic models for the 2009 Institute of Medicine (IOM) Guidelines and Chinese-recommended GWG which was made from this sample. The Chinese-recommended GWG was derived from the quartile values (25th-75th percentiles) of weight gain at the time of delivery in the subjects which comprised our sample.

**Results:**

For LBW children, using the recommended weight gain of the IOM and Chinese women as a reference, the OR for a pregnancy weight gain below recommendations resulted in a positive relationship for lean and normal weight women, but not for overweight and obese women. For macrosomia, considering the IOM’s recommended weight gain as a reference, the OR magnitude for pregnancy weight gain above recommendations resulted in a positive correlation for all women. The OR for a pregnancy weight gain below recommendations resulted in a negative relationship for normal BMI and lean women, but not for overweight and obese women based on the IOM recommendations, significant based on the recommended pregnancy weight gain for Chinese women. Of normal weight children, 56.6% were above the GWG based on IOM recommendations, but 26.97% of normal weight children were above the GWG based on Chinese recommendations.

**Conclusions:**

A GWG above IOM recommendations might not be helpful for Chinese women. We need unified criteria to classify adult BMI and to expand the sample size to improve representation and to elucidate the relationship between GWG and related outcomes for developing a Chinese GWG recommendation.

## Introduction

Gestational weight gain (GWG) is an important indicator to monitor and evaluate a pregnant woman’s nutritional status. In recent years, studies have shown that GWG is related to low birth weight (LBW) and macrosomia. In recent decades, the prevalence of macrosomia worldwide has been increasing to 4.7–13.1% [1–4]. In China, the incidence of macrosomia was 3.4–11.67% from 2005 to 2011 [5–8]. Macrosomia is associated with significant maternal and neonatal morbidity such as shoulder dystocia, caesarean birth, newborn asphyxia [6,9,10], and childhood obesity, as well as a high risk of cardiovascular diseases, diabetes, metabolic diseases, and obesity in adulthood [8].

Many studies reported that a high GWG was linearly correlated with macrosomia and excessive birth weight [11,12,13]. One cohort study reported that if a mother’s body mass index (BMI) increased by 25% or more during pregnancy, then 86.2% of the babies had macrosomia; thus, a high GWG was demonstrated to result in macrosomia [14].

LBW is one of the major causes of adverse prenatal outcomes and death. A study showed that neonatal death among infants weighing 1500–2500 grams is 20 times higher than that of normal weight infants; LBW is also a leading cause of childhood diseases and death [15]. The incidence of all LBW infants varied from 6.1% to 11% [16–19]. Papers concerning GWG and LBW indicated that underweight women, as well as women with a less-than-recommended GWG, were at a higher risk of delivering LBW babies [20,21].

Most studies that explored GWG and LBW children or children with macrosomia were based on populations in North America and Europe; fewer studies were conducted in developing countries. No suitable value exists regarding standard pregnancy weight gain in China, but adult BMI classification standards have been created in China [22]: <18.5 kg/m2 (lean), 18.5–24 kg/m2 (normal), 24–28 kg/m2 (overweight), and ≥28 kg/m2 (obese). The 2009 Institute of Medicine (IOM) Guidelines for GWG utilize standard body mass index (BMI) categories developed by the World Health Organization which is described as below: <18.5 kg/m2 (lean), 18.5–24.9 kg/m2 (normal), 25–29.9 kg/m2 (overweight), and ≥30 kg/m2 (obese) [23]. Obviously the Chinese adult BMI classification standards were lower than WHO BMI categories for normal, overweight and obese.

There was no official recommendation exist in China. It is necessary to elucidate the relationship between pregnancy weight gain and related outcomes by expanding the sample size to improve representative research and to develop an appropriate Chinese GWG value based on Chinese pre-pregnancy BMI.

Moreover, some studies restricted their analysis to either pre-pregnancy weight gain and pregnancy weight gain or correlations between maternal weight parameters and mean birth weight, without estimating the relative risk of macrosomia and LBW [24–27].

Thus, this large cohort study used adult Chinese BMI classification standards and IOM-recommended GWGs to assess whether the relationship between GWG and LBW children or children born with macrosomia was modified by pre-pregnancy BMI, and estimates of the relative risk of macrosomia and LBW based on pre-pregnancy BMI were controlled.

## Materials and Methods

### Study population

Our study was conducted under the approval of the Ethics Committee of Wuhan Women and Children Health Care Center and Tongji Medical College of Huazhong University of Science and Technology. At recruitment, written informed consent was obtained from parents of the subjects.

Wuhan is a large city with over 10.12 million residents in the middle of China. Further, more than 4.8 million residents live in seven inner districts. Prenatal care and child health care information in Wuhan were collected from a comprehensive perinatal health care information system that was established to improve perinatal outcome surveillance by Wuhan Medical and Health Center for Women and Children (MHCWC) two decades ago. This system consists of maternal/infant health care centers at three levels: city (MHCWC), district, and community. One of the major tasks of the system is to conduct surveillance of pregnancy outcomes. All pregnant women are required to register at their district maternal health care center within three months of becoming pregnant.

In general, during the first prenatal care visit, each pregnant woman receives a manual with instructions for prenatal and postnatal care; she is also given forms for obstetricians to record data on maternal age, height before pregnancy, weight before pregnancy, maternal education, and smoking during pregnancy. Additionally, she receives a complete physical examination including an ultrasound examination. All childbirth information such as infant gender and birth weight and pre-delivery maternal weight were collected within hospital information systems. After delivery, mothers were required to visit their local maternal health care centers. During the visit, health care workers arranged a series of postnatal visits based on information recorded in the returned manual. In addition, the perinatal health care system requires that health care workers at community maternal/infant health care centers visit a woman within 42 days of her delivery. The regionalized perinatal health care system in Wuhan enables almost complete follow-up of all women.

Strict and standardized QA/QC procedures are utilized: birth delivery data is validated four times a year; computerized data is examined against the original records at delivery hospitals; each living newborn infant must be registered at a community maternal/infant health care center; birth delivery information is updated every month including confirmation of newborn infants’ name, gender, date and time of birth, and parental names; four to five randomly selected community maternal/infant health care centers are chosen for data audits in each of the seven districts every year; and the MHCWC is responsible for training the district center health care workers responsible for training community center health care workers.

All health data for both pregnant women and their children in this study were collected and available from the perinatal health care system from June 30, 2011 to June 30, 2013. The sample criteria were children born during this period, mothers living in the inner city districts of Wuhan, a gestational age of ≥28 weeks, a singleton, and live birth. The total sample size was 85,765.

### Measurements

Specially trained gynecologists measured birth weight and maternal weight before delivery on a platform scale (RGZ-12-RT, WuXi Weighing Apparatus Co) in primary hospitals. Birth weight was measured to the nearest 0.01 kg using a digital scale (HCS-20/30-YE, TaiXing Weighing Apparatus Co). One validity study was conducted to compare electronically measured data (birth weight and weight before delivery) and the hospitals’ measurements (birth weight and pre-delivery weight) of 875 children from seven major hospitals. The correlation between these two measurements was 0.990.

Pre-pregnancy BMI was calculated as follows: weight/height2. Pre-pregnancy BMI was categorized into four groups that are standard in China [22]: <18.5 kg/m2 (lean), 18.5–24 kg/m2 (normal), 24–28 kg/m2 (overweight), and ≥28 kg/m2 (obese). According to the IOM recommendations, we defined adequate weight gain as follows: 12.5–18 kg (lean), 11.5–16 kg (normal), 7–11.5 kg (overweight), and at least 5–9 kg (obese).

The recommended weight gain in Chinese women was derived from the quartile values (25th-75th percentiles) of weight gain at the time of delivery in the 76,854 subjects which comprised our sample. To determine the normative distribution of weight gain, subjects with good pregnancy outcomes were identified. A good outcome was defined as a delivery at term (between 37 and 42 weeks gestation) of a live infant with a birth weight between 2500 and 4000 g to a mother without prenatal complications, such as diabetes or hypertension. Table 1 shows the total maternal weight gain in Chinese women with different BMIs. The differences between IOM and Chinese-recommended weight gain by BMI Categories were described in Table 2.

**Table 1:** Total maternal weight gain in Chinese women of different pre pregnancy BMI.

Prepregnancy BMI	China BMI Standard (Kg/m2)	No. of Subjects(%)	Total weight gain (kg),mean±SD*	25th percentile(kg)	75th percentile(kg)	10th and 90 th percentiles(kg)
Underweight	<18.5	13223(17.21)	19.0±6.3	15	22	12.0–27.0
Average	18.5–23.9	58765(76.46)	17.2±7.1	13	21	9.0–26.0
Overweight	24–27.9	4256 (5.54)	14.4±6.2	10	18	6.0–22.0
obese	>28	610 (0.79)	13.5±5.9	9.5	17	5.0–21.0

**Table 2:** The differences between IOM and Chinese-recommended weight gain by BMI Categories.

Prepregnacy BMI	China BMI Standard (Kg/m2)	Chinese-recommended Weight Gain	IOM BMI Standard (kg/ m2)	IOM-recommended Weight Gain
Underweight	<18.5	15.0–22.0	<18.5	12.5–18.0
Average	18.5–23.9	13.0–21.0	18.5–24.9	11.5–16.0
Overweight	24–27.9	10.0–18.0	25.0–29.9	7.0–11.5
Obese	>28	9.5–17.0	>30.0	5.0–9.0

As defined by the WHO, birth weight was divided into low birth weight (<2500 g), normal birth weight (2500–4000 g), and macrosomia (birth weight ≥ 4000 g).

### Statistical analysis

Several potential covariates were chosen according to previous studies, including maternal age, education, infant gender, and smoking during pregnancy. All data came from the prenatal health care system. Frequencies were calculated to describe birth weight, pre-pregnancy BMI, gestational weight gain, and potential covariates. Linear regression was used to evaluate the association between mean birth weight and pre-delivery BMI. A scatter diagram of birth weight and weight gain was described.

We used a stratified analysis by pre-pregnancy BMI groups. Logistic regression models were used to estimate the independent association between pregnancy weight gain, LBW, normal birth weight, and macrosomia within different pre-pregnancy BMI groups. We built different logistic models for IOM- and Chinese-recommended GWG. Each logistic regression model was adjusted for potential covariates. Crude and adjusted overall risk (OR) statistics and the 95% confidence interval (CI) were calculated. For comparison purposes, all models include the same covariates.

All statistical analyses were performed using SPSS software (Statistics 20, SPSS, USA).

## Results

The subjects’ demographic characteristics are shown in Table 3. The mean birth weight was 3313.90 g; 2.9% of the singleton infants had a LBW; and the incidence of macrosomia was 6.5%. The women’s pre-pregnancy BMIs were as follows: 16.9% were lean, while overweight and obese were 6.7%. The mean pre-pregnancy BMI was 20.41 ± 2.27. The pre-delivery BMI was 27.15 ± 0.01. The mean weight gain was 17.39 ± 7.22; a large proportion of the women (57.5%) had a pregnancy weight gain above the IOM recommendations. Further, 51.5% of the women were 25–29 years old, and 11.7% had obtained a junior high school education or less. Meanwhile, 53.2% of the infants were male. Almost none of the pregnant women smoked during pregnancy.

**Table 3:** Characteristics of women and infant.

Characteristics	N (%)
**Birth weight**	
Low birth weight	2500(2.9)
Normal birth weight	77674(90.6)
Macrosomic	5591(6.5)
**Pre pregnant BMI**	
Lean	14477(16.9)
Normal	65536(76.4)
Overweight and obese	5752(6.7)
**Weight gain**	
Below IOM	14985(17.5)
Wthin IOM	21461(25.0)
Above IOM	49319(57.5)
**Age**	27.92±4.059
≤19	829(1.0)
20–24	15360(17.9)
25–29	44151(51.5)
30–34	19823(23.1)
≥35	5602(6.5)
**Education**	
Elementary school	1477(1.7)
Junior high school	8547(10.0)
Senior high school	38479(44.9)
College or graduate school	37144(43.4)
**Infants Gender**	
Male	45667(53.2)
Female	40098(46.8)
**Smoking during pregnancy**	
No	85765(100.0)

**Fig 1:**
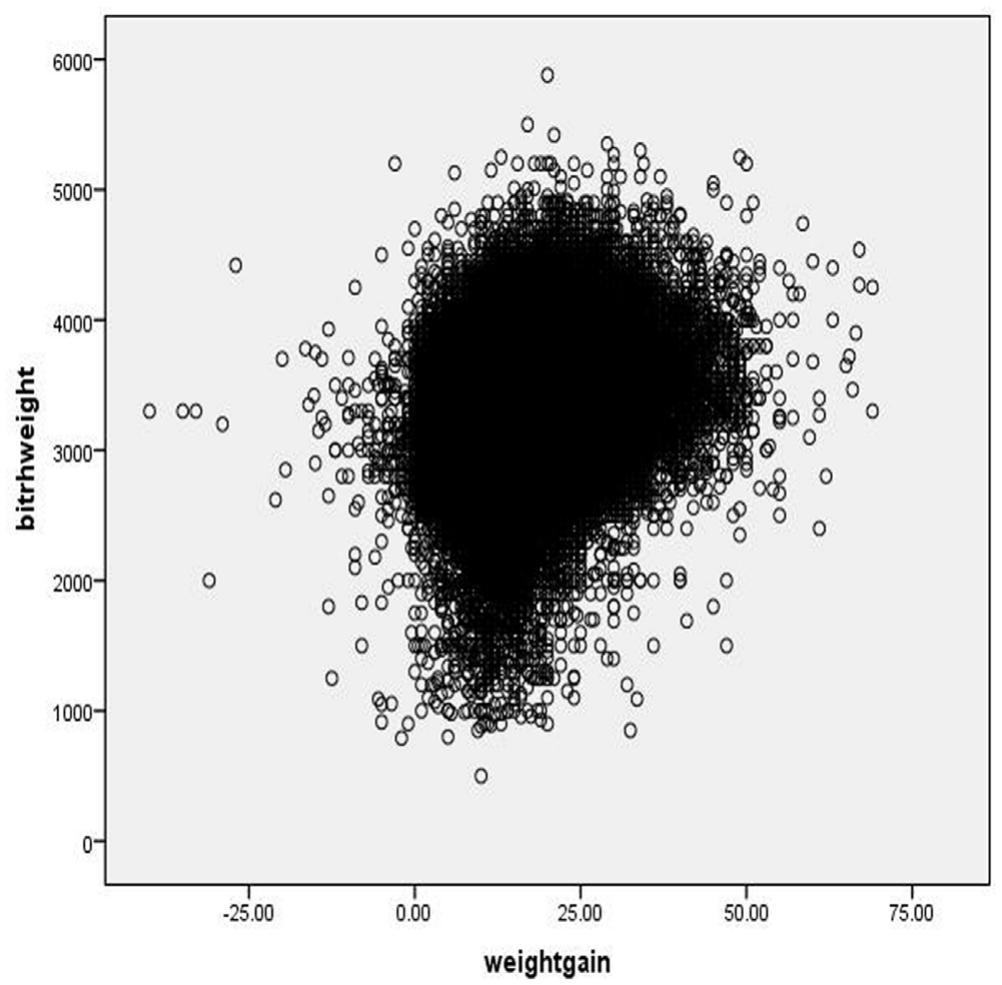
Scatter diagram of birth weight and weight gain.

A correlation analysis showed a significant but weak correlation between the mean birth weight and weight gain, as shown in Fig 1 (r = 0.240; P < 0.001).

The ORs for LBW by weight gain and pre-pregnancy BMI and OR for macrosomia are described in Tables 4 and 5. Pre-pregnancy BMI modified both the association between IOM-recommended pregnancy weight gain and LBW and Chinese-recommended pregnancy weight gain and LBW. For LBW children, considering the IOM’s recommended weight gain and the Chinese recommended pregnancy weight gain as a reference, the OR magnitude for a pregnancy weight gain above recommendations resulted in a negative relationship for normal BMI women (IOM: OR = 0.454, CI = 0.404–0.510; Chinese: OR = 0.389, CI = 0.331–0.456) and overweight and obese women (IOM: OR = 0.508, CI = 0.380–0.679; Chinese: OR = 0.268, CI = 0.169–0.426), lean women (IOM: OR = 0.310, CI = 0.244–0.394; Chinese: OR = 0.457, CI = 0.333–0.627).

**Table 4:** ORs for LBW by weight gain and pre pregnant BMI.

Pre pregnant BMI (Kg/m^2^)	LBW (Yes/No)		Crude OR (95%CI)	P	Adjusted OR(95%CI)*	Adjusted P*
**Lean(<18)**						
**Weight gain**						
Below	156/1585	IOM	2.132(1.723,2.638)	<0.0001	2.141(1.730,2.650)	<0.0001
	239/3143	Chinese	2.664(2.189,3.240)	<0.0001	2.652(2.179,3.227)	<0.0001
Within	217/4723	IOM ref.				
	187/6550	Chinese ref.				
Above	102/7139	IOM	0.309(0.243,0.392)	<0.0001	0.310(0.244,0.394)	<0.0001
	49/3754	Chinese	0.457(0.333,0.628)	<0.0001	0.457(0.333,0.627)	<0.0001
**Normal(18–24)**						
**Weight gain**						
Below	605/11627	IOM	1.285(1.143,1.444)	<0.0001	1.277(1.135,1.436)	<0.0001
	737/14956	Chinese	1.626(1.471,1.798)	<0.0001	1.621(1.466,1.793)	<0.0001
Within	568/14103	IOM ref.				
	863/28480	Chinese ref.				
Above	619/33643	IOM	0.456(0.407,0.512)	<0.0001	0.454(0.404,0.510)	<0.0001
	189/15837	Chinese	0.394(0.336,0.462)	<0.0001	0.389(0.331,0.456)	<0.0001
**Over weight and obese(>24)**						
**Weight gain**						
Below	30/573	IOM	0.698(0.458,1.066)	0.096	0.700(0.458,1.067)	0.0975
	74/1145	Chinese	1.123(0.840,1.500)	0.4346	1.123(0.840,1.500)	0.4344
Within	73/982	IOM ref.				
	141/2449	Chinese ref.				
Above	130/3299	IOM	0.508(0.380,0.679)	<0.0001	0.508(0.380,0.679)	<0.0001
	21/1360	Chinese	0.268(0.169,0.426)	<0.0001	0.268(0.169,0.426)	<0.0001

*Adjusted for maternal age, maternal education, infant gender. Smoking was not one confounding factor because of no smoking women.

**Table 5:** ORs for macrosomic by weight gain and pre pregnant BMI.

Pre pregnant BMI (Kg/m^2^)	Macrosomic (Yes/No)		Crude OR (95%CI)	P	Adjusted OR(95%CI)*	Adjusted P*
**Lean(<18)**						
**Weight gain**						
Below	13/1585	IOM	0.602(0.366,0.989)	0.0452	0.602(0.366,0.989)	0.0451
	35/3143	Chinese	0.346(0.241,0.496)	<0.0001	0.346(0.241,0.496)	<0.0001
Within	93/4723	IOM ref.				
	211/6550	Chinese ref.				
Above	449/7139	IOM	3.158(2.518,3.961)	<0.0001	3.159(2.519,3.962)	<0.0001
	309/3754	Chinese	2.555(2.135,3.058)	<0.0001	2.553(2.134,3.056)	<0.0001
**Normal(18–24)**						
**Weight gain**						
Below	361/11627	IOM	0.750(0.658,0.853)	<0.0001	0.750(0.659,0.854)	<0.0001
	493/14956	Chinese	0.567(0.512,0.628)	<0.0001	0.568(0.513,0.629)	<0.0001
Within	623/14103	IOM ref.				
	1665/28480	Chinese ref.				
Above	3387/33647	IOM	2.269(2.078,2.477)	<0.0001	2.264(2.074,2.472)	<0.0001
	2209/15837	Chinese	2.400(2.264,2.566)	<0.0001	2.396(2.241,2.561)	<0.0001
**Over weight and obese(>24)**						
**Weight gain**						
Below	35/573	IOM	0.686(0.463,1.017)	0.0607	0.687(0.464,1.019)	0.0618
	55/916	Chinese	0.538(0.415,0.698)	<0.0001	0.540(0.417,0.700)	<0.0001
Within	79/982	IOM ref.				
	306/2449	Chinese ref.				
Above	551/3299	IOM	1.895(1.505,2.386)	<0.0001	1.893(1.503,2.384)	<0.0001
	296/1360	Chinese	1.742(1.465,2.071)	<0.0001	1.747(1.470,2.078)	<0.0001

*Adjusted for maternal age, maternal education, infant gender. Smoking was not one confounding factor because of no smoking women.

Using the recommended weight gain of the IOM and Chinese women as a reference, the OR for a pregnancy weight gain below recommendations resulted in a positive relationship for women with normal BMIs (IOM: OR = 1.277, CI = 1.135–1.436; Chinese: OR = 1.621, CI = 1.466–1.793) and lean women (IOM: OR = 2.141, CI = 1.730–2.650; Chinese: OR = 2.652, CI = 2.179–3.227), but not for overweight and obese women (IOM: OR = 0.700, CI = 0.458–1.067; Chinese: OR = 1.123, CI = 0.840–1.500). The pre-pregnancy BMI modified the association between the IOM recommended pregnancy weight gain, the Chinese recommended pregnancy weight gain, and macrosomia. For macrosomia, considering the IOM’s recommended weight gain as a reference, the OR magnitude for pregnancy weight gain above recommendations resulted in a positive correlation for lean (IOM: OR = 3.159, CI = 2.519–3.962; Chinese: OR = 2.553, CI = 2.134–3.056), normal weight (IOM: OR = 2.264, CI = 2.074–2.472; Chinese: OR = 2.396, CI = 2.241–2.561), and overweight and obese women(IOM: OR = 1.893, CI = 1.503–2.384; Chinese: OR = 1.747, CI = 1.470–2.078).

Using the recommended weight gain of the IOM and Chinese women as a reference, the OR for a pregnancy weight gain below recommendations resulted in a negative relationship for women with a normal BMI (IOM: OR = 0.750, CI = 0.659–0.854; Chinese: OR = 0.568, CI = 0.513–0.629) and lean women (IOM: OR = 0.602, CI = 0.366–0.989; Chinese: OR = 0.346, CI = 0.241–0.496). In overweight and obese women, the relationship was not significant based on the IOM recommendations (OR = 0.687, CI = 0.464–1.019), but significant based on the recommended pregnancy weight gain for Chinese women (OR = 0.540, CI = 0.417–0.700).

Of normal weight children, 56.6% were above the GWG based on IOM recommendations, but 26.97% of normal weight children were above the GWG based on Chinese recommendations. Of LBW infants, 42% were below the GWG based on Chinese recommendations and 31.8% of LBW infants were below the GWG based on IOM recommendations. Of macrosomic infants, 50.33% were above the GWG based on Chinese recommendations and 77.71% of macrosomic infants were below the GWG based on IOM recommendations. Table 6 shows the weight gain based on IOM and Chinese recommendations.

**Table 6:** Cross table of weight gain by IOM and Chinese.

	Weight Gain recommendation (IOM) N (%)			Weight gain recommendation (Chinese) N (%)		
	Below	Within	Above	Below	Within	Above
Normal birth weight	13850(17.83)	19858(25.57)	43966(56.60)	19244(24.78)	37479(48.25)	20951(26.97)
Low birth weight	792(31.68)	864(34.56)	844(33.76)	1050(42.00)	1191(47.64)	259(10.36)
Macrosomic	440(7.87)	806(14.42)	4345(77.71)	605(10.82)	2172(38.85)	2814(50.33)

## Discussion

In this large population-based study of pregnant Chinese women, we highlighted the relationship between weight gain and birth weight that was modified by pre-pregnancy BMI.

### Comparison with previous reports

Weight gain above IOM and Chinese recommendations had a significant, negative correlation with LBW for normal weight, overweight, obese, and lean women. A previous study indicated that the severity of low pre-pregnancy BMI was associated directly (in a dose-response fashion) with preterm birth and LBW [28]. Our study indicated that for lean women, compared to women within the recommended GWG, weight gain less than the recommended GWG increased the risk of LBW. Weight gain greater than the recommended GWG decreased the risk of LBW. For overweight and obese women, compared to women within the recommended GWG, we did not demonstrate a relationship between women within the recommended GWG, greater than the recommended GWG, and LBW.

We found that weight gain above IOM and Chinese recommendations was associated with macrosomia and demonstrated a positive correlation for lean (weight gain > 18 kg), normal (weight gain > 16 kg), and overweight and obese women (weight gain, >11.5 kg and >9 kg, respectively). This result indicated that a large gestational weight gain equated to a high risk of macrosomia that was consistent with several studies [5,6].

Weight gain below IOM and Chinese recommendations was significant for macrosomia. The result was similar to other studies that demonstrated a weight gain below IOM recommendations was inversely related to macrosomia [29] or associated with a more favorable birth weight for all obese women [30]. The relationship between weight gain below IOM or Chinese recommendations and LBW was significant that was consistent with previous studies [31]. Another study reported that lean mothers or those who gained less weight than recommended had twice the risk of delivering LBW babies, as compared with women who had a normal BMI [32].

### The need for a new weight gain guideline for Chinese women

We found that 56.6% of normal weight children were above the GWG based on IOM recommendations, but 26.97% of normal weight children were above the GWG based on Chinese recommendations. Our study result suggested that a GWG above IOM recommendations might not be helpful for Chinese women. The data which were used to make the IOM guidelines may be old or not used in Asian countries. One study in Thailand reported that poor maternal weight gain during pregnancy was associated with SGA (small for gestational age) infants and LBW infants. However, as compared with Thai guidelines, the 2009 IOM guidelines were not sensitive if attempting to predict SGA infants.

Over the past 50 years, gestational weight gain recommendations have always been controversial worldwide. In 1990, the IOM made novel recommendations. It set different weight gain standards that were based on pre-pregnancy BMI that encouraged normal weight women to gain 25–35 lb, while lean women were to gain more and overweight and obese women were to gain less. However, research indicated a higher target for pregnancy weight gain would result in a higher percentage of macrosomic infants [33], so they created new guidance in 2009 that reduced the weight gain recommendation for obese women to 5–9 kg. The IOM used data that comprised white women in industrialized countries to assess gestational weight gain. Thus, the IOM guide was often used in Europe and the United States. Much research has focused on pre-pregnancy BMI, GWG, and various pregnancy outcomes, as well as appropriate weight management for local populations to put forth GWG guidance for their own countries. At present, we have not yet created an official GWG recommendation in China. Previous Chinese studies thought the 2009 IOM GWG recommendation might be suited to a Chinese population [34]. However, prolonged production stagnation, NICU neonatal transfer, and forceps delivery risk resulted in no difference in appropriate GWG and insufficient and excessive GWG. It necessitates further study to determine if the same result would occur in a small sample.

A study recommended a different GWG for different cities [35]. These studies obtained samples from China, and the researchers proposed different GWG recommendations. The difference may be related to the study population, BMI classification criteria, and research methods. Currently, adult BMI classification criteria presented by the Chinese people obesity and disease risk Collaborative Group that have high scientific standards and reliability are widely used, including underweight, normal, overweight, and obesity BMI criteria that are defined as <18.5, 18.5–23.9, 24.0–27.9, and >28.0, respectively [22].

### Limitations

Our study had several limitations. We did not obtain samples from the rural districts of Wuhan. Pregnant women who live in rural districts may have a different weight status than women who live in the inner city districts of China; therefore, the results are not nationally representative and may not be generalizable to all women. The secondhand smoke information which there are high levels of exposure in China was not collected. That may be associated with low birth weight. The patients reported their own pre-pregnancy weight and height to the obstetricians that may have led to misclassification in the present study. It is possible that heavier women underreported their pre-pregnancy weight.

## Conclusion

To conclude, a great amount of GWG may lead to a high risk of macrosomic and reduce the risk of LBW. There was clear association with weight gain below IOM recommendations for macrosomia and LBW. Many more normal weight children were above the GWG based on IOM recommendations, but fewer normal weight children were above the GWG based on Chinese recommendations. That result may indicate that we need unified criteria to classify adult BMI and to expand the sample size to improve representation and to elucidate the relationship between GWG and related outcomes for developing a Chinese GWG recommendation.
